# The altered T cell landscape in systemic sclerosis patients is characterized by dysfunctional type 1 immunity

**DOI:** 10.1093/jimmun/vkag288

**Published:** 2026-11-04

**Authors:** Victoria Volfson-Sedletsky, Hannah A DeBerg, Mitch L Fahning, Ian R Rifkin, Andreea M Bujor, Daniel J Campbell, Peter A Morawski, Anna C Belkina, Hans Dooms

**Affiliations:** Arthritis and Autoimmune Diseases Center, Rheumatology Section, Department of Medicine, Chobanian and Avedisian School of Medicine, Boston University, Boston, MA, United States; Department of Virology, Immunology and Microbiology, Chobanian and Avedisian School of Medicine, Boston University, Boston, MA, United States; Center for Systems Immunology, Benaroya Research Institute, Seattle, WA, United States; Center for Fundamental Immunology, Benaroya Research Institute, Seattle, WA, United States; Renal Section, Department of Medicine, Boston Medical Center, Boston, MA, United States; Renal Section, Department of Medicine, VA Boston Healthcare System, Boston, MA, United States; Arthritis and Autoimmune Diseases Center, Rheumatology Section, Department of Medicine, Chobanian and Avedisian School of Medicine, Boston University, Boston, MA, United States; Center for Fundamental Immunology, Benaroya Research Institute, Seattle, WA, United States; Department of Immunology, University of Washington School of Medicine, Seattle, WA, United States; Center for Fundamental Immunology, Benaroya Research Institute, Seattle, WA, United States; Department of Virology, Immunology and Microbiology, Chobanian and Avedisian School of Medicine, Boston University, Boston, MA, United States; Department of Pathology, Chobanian and Avedisian School of Medicine, Boston University, Boston, MA, United States; Arthritis and Autoimmune Diseases Center, Rheumatology Section, Department of Medicine, Chobanian and Avedisian School of Medicine, Boston University, Boston, MA, United States; Department of Virology, Immunology and Microbiology, Chobanian and Avedisian School of Medicine, Boston University, Boston, MA, United States; Theodore and Maxine Murnick Family Department of Immunology and Genomic Medicine, National Jewish Health, Denver, CO, United States; Department of Immunology and Microbiology, University of Colorado Anschutz Medical Campus, Aurora, CO, United States

**Keywords:** fibrosis, IFN-γ, systemic lupus erythematosus, systemic sclerosis, type 1 T cells

## Abstract

T cells in patients with chronic autoimmunity show features of dysfunction. However, the functionally defined T cell subsets that are affected by these changes remain poorly characterized. Here, we sought to reveal aberrations in the composition, phenotype, and function of canonical T cell subsets in the blood of systemic sclerosis (SSc) patients compared with healthy subjects and systemic lupus erythematosus (SLE) patients. Therefore, we developed a novel multidimensional flow-cytometry panel to simultaneously detect lineage-defining transcription factors, co-inhibitory receptors and other functional markers to characterize T cell subsets without in vitro restimulation. We compared T cell landscapes in SSc and SLE patients, and healthy subjects, using opt-SNE (optimized t-distributed stochastic neighbor embedding) and PhenoGraph algorithms. The data show altered distribution and aberrant functional states of transcription factor–defined T cell subsets in patients with autoimmunity, with shared and unique features between diseases and their subtypes. Strikingly, SSc and SLE patients showed deficiencies in subsets of CD8^+^ and CD4^−^CD8^−^ T cells that expressed T-bet, which is critical for IFN-γ–dependent type 1 immunity. Moreover, TIGIT^+^Foxp3^+^ regulatory T cells, which suppress T helper 1 responses, were increased in diffuse cutaneous SSc and SLE patients. Functionally, T cells from SSc patients produced less IFN-γ, a cytokine that suppresses profibrotic gene expression in fibroblasts, as we demonstrate by transcriptomic analysis. Our study supports a model in which IFN-γ opposes canonical fibrotic gene programs in fibroblasts, and deficiencies in the IFN-γ–producing T cell compartment in SSc patients therefore may contribute to chronic fibrosis development. Aberrant T cell subsets in SSc and SLE represent targets for corrective immunotherapy.

## Introduction

Systemic sclerosis (SSc) or scleroderma is a complex autoimmune disease characterized by immune dysregulation, vasculopathy, and progressive fibrosis of the skin and internal organs. While the precise role of T cells in SSc pathogenesis remains incompletely understood, a growing body of evidence implicates their involvement in promoting disease pathology.^1^^,^^2^ Multiple T cell subsets, including T helper 1 (Th1), Th2, Th17, regulatory T cells (Tregs), T follicular helper (Tfh), cytotoxic CD4^+^ T cells, and CD8^+^ T cells have been implicated in the disease process.^3–9^ The role of these T cell subsets in fibrosis may be attributable to their production of pro- and antifibrotic cytokines, cytotoxic activities, immune regulation, and help with autoantibody generation. Many of these functions are regulated by costimulatory (co-SR) and co-inhibitory (co-IR) receptors. We and others have shown that some co-IRs are increased on CD4^+^ and CD8^+^ T cell subsets in SSc,^10^ as well as in many other autoimmune diseases.^11–14^ However, a detailed analysis of the functionally defined T cell lineages and their subsets that are affected by specific patterns of co-IR and co-SR expression has not been performed. One challenge to link co-SR and co-IR expression to functional subsets is the need to restimulate T cells in vitro to define their cytokine production profiles. This process modulates co-SR/co-IR expression, making assignment of the phenotypes to the original ex vivo subsets potentially inaccurate. To address this problem, we designed a novel multidimensional spectral flow cytometry panel that combines staining for transcription factors, defining cytokine-competent T cell lineages, with various surface and intracellular markers indicative of T cell fitness and functional state. This approach allowed us to directly quantify the presence of functionally defined CD4^+^, CD8^+^, and CD4^−^CD8^−^ T cell subsets in peripheral blood of patients with diffuse cutaneous SSc (dcSSc), limited cutaneous SSc (lcSSc), and SLE, and define their phenotype. We hypothesized that SSc patients would exhibit changes in the frequencies and phenotypes of profibrotic as well as antifibrotic T cell subsets. Our study revealed significant differences in the T cell landscape of SSc patients, SLE patients, and healthy subjects, mainly characterized by a striking defect of specific T-bet–expressing T cell populations in SSc and SLE. Because T-bet–expressing T cells are the main producers of the canonical type 1 cytokine IFN-γ and are protective in fibrosis models,^15–17^ their deficiency in frequency and function may significantly impact SSc disease course. In support of this, we show that memory T cell subsets from SSc patients produced less IFN-γ after in vitro restimulation, and we confirm that IFN-γ suppresses profibrotic gene expression in fibroblasts.^18^^,^^19^ We also found an increase in TIGIT-expressing Tregs that may further suppress IFN-γ–producing T cells.^20^ Hence, evidence from our 3 independent experimental approaches, namely immune phenotyping, cytokine assays, and transcriptomic analysis of fibroblasts, suggests that a defective IFN-γ compartment may contribute to weakened antifibrotic activities in SSc patients.

## Methods

### Sex as a biological variable

Our study examined both male and female tissues but was not powered to detect sex-based differences.

### Study participants

Blood samples were obtained from patients in BD Vacutainer CPT with citrate or EDTA Blood Collection Tubes (BD Vacutainer). All SSc patients fulfilled the 2013 revised American College of Rheumatology classification criteria.^21^ Patients were classified as having dcSSc or lcSSc disease according to LeRoy criteria,^22^ and were sex, race, and age matched with healthy subjects. Diagnosis of SLE was made following European Alliance of Associations for Rheumatology/American College of Rheumatology criteria.^23^ Some patients were treated with 1 or more immunosuppressive drugs (CellCept, methotrexate, tofacitinib, prednisone, and hydroxychloroquine) at the time of blood draw.

### Peripheral blood mononuclear cell isolation

Blood samples were collected and peripheral blood mononuclear cell (PBMCs) isolated the same day or up to 18 h post–blood collection. PBMCs were isolated either by underlaying with Lymphoprep (StemCell Technologies) and separating PBMCs by density dependent centrifugation, or by using a BD Vacutainer CPT with citrate. Prior studies have shown that CPT- and Ficoll-isolated PBMCs are broadly comparable with respect to major immune subset composition and key functional readouts.^24^^,^^25^ PBMC samples were frozen in 50% complete RPMI 1640 medium (10% fetal bovine serum [FBS], 1% nonessential amino acids, 1% HEPES, 1% penicillin-streptomycin, 1% sodium pyruvate, 0.1% β-mercaptoethanol) with 40% FBS and 10% dimethyl sulfoxide using a Mr. Frosty freezing container and placed overnight at −80 °C before transferring to a liquid nitrogen tank.

### High-dimensional multiparameter full spectrum flow cytometry and algorithmic analysis

For staining, frozen PBMCs were thawed in a 37 °C water bath for 2 min. Cells were resuspended in complete RPMI 1640 medium and centrifuged at 300 *g* for 10 min at 4 °C. Supernatant was discarded and cells were resuspended in medium and washed. Next, thawed PBMCs were washed in phosphate-buffered saline (PBS) and stained with Live/Dead stain (UV zombie, 1:800), following the manufacturer’s instructions (BioLegend). For staining of cell surface markers, cells were resuspended in PBS + 2% FBS and Fc receptors blocked with TruStain FcX (BioLegend), followed by addition of the fluorescently labeled antibodies specified in Table S1A. All antibody clones and concentrations were extensively tested and optimized using methods we previously published.^26^ To minimize batch effects, antibody lots were prevalidated using reference samples, and titrations were adjusted when needed to reduce lot-to-lot variation in signal intensity. Samples were stained for 30 min in the dark at 4 °C. Excess antibody was removed by washing in PBS + 2% FBS. For subsequent intracellular staining, cells were fixed in Foxp3 Fixation/Permeabilization working solution (eBioscience) for 1 h at room temperature in the dark. Cells were washed with permeabilization buffer, and intracellular antibody mix was added to the samples and incubated for 2 h at room temperature in the dark. Cells were washed twice with permeabilization buffer and resuspended in PBS + 2% FBS before being analyzed. High-dimensional multiparameter full-spectrum flow cytometry was performed on the 5 laser Cytek Aurora analyzer (Cytek Biosciences). For spectral unmixing, UltraComp compensation beads (Thermo Fisher Scientific) or single-stained PBMCs were used, and the data analyzed with SpectroFlo (Cytek Biosciences) software, using the ordinary least square algorithm. A data processing pipeline was established using the Omiq.ai cloud computation platform. Live single-cell lymphocytes were manually gated on CD3 followed by manual gating of CD4^+^, CD8^+^, and CD4^−^CD8^−^ T cells. Unmixed spectral cytometry data (FCS files) were uploaded into omiq, data were arcsinh-transformed (cofactor: 6,000), and scales were adjusted per signal range for each feature. All channel measurements, except CD3, CD4, CD8, PD-1, Vδ1, and Vδ2, were projected into 2-dimensional space using the optimized t-distributed stochastic neighbor embedding (opt-SNE) algorithm.^27^

Data were acquired in 9 batches totaling 80 PBMC samples, with 75 samples included in the final analysis after quality control filtering. Within each cohort (healthy control subjects, SSc patients, SLE patients), samples were distributed across batches to prevent group-specific bias. To further minimize batch effects, marker distributions were evaluated across experimental batches to identify batch-associated shifts potentially arising from reagent variation or unmixing artifacts. Of all measured features, only BV421 PD-1 showed a consistent batch-associated shift in median fluorescence intensity that could not be adequately corrected by normalization approaches, including CytoNorm. Because inclusion of this feature caused samples to separate by batch and interfered with biologically meaningful unsupervised clustering, PD-1 was excluded from dimensionality reduction and clustering analyses. PD-1 expression was nevertheless evaluated within individual batches using batch-specific fluorescence-minus-one controls and by conventional manual gating. T cell receptor Vδ1 and Vδ2 staining was not included in the initial batches and therefore also not in the input for automated clustering algorithm.

The following parameters of the algorithm were used: random initialization, perplexity = 40, KLD minimization endpoint = 5,000. For PhenoGraph, which clusters cell populations in an unbiased manner based on their expression profiles of all the markers of interest, the following parameters were used: kNN = 20; distance: Euclidean; features for clustering: TIGIT, T-bet, CXCR5, CD226, RORγt, Ki-67, Foxp3, Bcl-6, GATA-3, TOX, CD45RO. After the unsupervised clustering step, datapoints for each cluster were visualized as heatmap overlays over opt-SNE maps. To assure that the different cohorts in our dataset (healthy subjects, dcSSc patients, lcSSc patients, and SLE patients) showed comparable overall cell distribution patterns, opt-SNE maps were overlayed with cell densities for each cohort for all 3 T cell lineages (Fig. S1). Major differences in cell distribution and cell population ratios between the groups may indicate technical issues and can result in an inaccurate analysis, both biological and statistical. Each cohort contained an equal number of CD4^+^ (1 × 10^6^ cells), CD8^+^ (1 × 10^6^ cells), and CD4^−^CD8^−^ (250,000) T cells. We did not observe large regions that were only present in some cohorts but not in others, indicating that the stained samples were intact and the staining quality comparable, allowing us to execute analysis of all 4 cohorts. PhenoGraph-identified clusters were validated and refined through conventional manual expert gating based on two criteria: (i) minor batch-to-batch variations in signal and expected algorithm overclustering were addressed by combining highly similar populations that had been assigned to different clusters and (ii) heterogeneous populations clustered together due to weak signal from some markers were manually separated to maintain biological validity. Final cluster assignments were verified by overlaying cluster designations on opt-SNE maps to confirm spatial coherence and biologically expected marker coexpression patterns. Frequencies of individual clusters were calculated for each subject (all data available as Supplementary Material), and data were plotted using Prism 8.0 (GraphPad Software). Finally, to validate the robustness of our automated clustering approach, we confirmed key findings using conventional manual gating on bivariate flow cytometry plots. Cluster-defined populations were verified by setting gates on well-established T cell lineage markers using single-batch fluorescence-minus-one controls and standard gating strategies. Results from manual gating were concordant with PhenoGraph-identified subsets, and frequencies remained consistent with expected immune cell subsets and published literature (e.g. expected frequency of Foxp3+ Tregs within healthy control subjects, etc.), confirming that our conclusions are not algorithm-generated artifacts.

### T cell intracellular cytokine staining

Frozen PBMCs were thawed, washed with wash buffer (RPMI + 3% FBS), resuspended in ImmunoCult-XF T Cell Expansion Medium (StemCell Technologies; 10981), and rested overnight. The next morning, untouched T cells were isolated using pan-T cell magnetic beads (Miltenyi Biotec; 130-096-535). T cells were stimulated with PMA (50 ng/mL; Sigma-Aldrich; P8139) and ionomycin (1 μg/mL; Sigma-Aldrich; I06434) with brefeldin A (10 μg/mL; Sigma-Aldrich; B6542) for 5 h. Surface staining was performed for 15 min at 37 °C with antibodies diluted in 0.3% bovine serum albumin–Hanks’ buffered salt solution (Table S1B). For permeabilization and fixation, Cytofix/Cytoperm was used (Becton Dickinson; RUO 554714). Intracellular cytokine staining was performed for 30 min at 4 °C. Data were acquired on LSRFortessa or LSRII, and analyzed using FlowJo software (v.10.9; Becton Dickinson).

### Stimulation of fibroblasts, bulk RNA sequencing, and bioinformatic analysis

Human dermal fibroblasts from the eyelid of a single healthy donor were purchased from a commercial vendor (ZenBio; DF-F) and cultured at low passages (*P* = 5) using Dulbecco’s Modified Eagle Medium supplemented with 10% FBS, 2 mM L-glutamine, 100 U/mL penicillin, and 100 mg/mL streptomycin (Gibco; 10938025). Fibroblasts, approximately 1 to 2 × 10^4^ in a 96-well flat-bottom plate, were treated for 24 h with carrier-free recombinant human IFN-γ, IL-13, or IL-17a (BioLegend; 570202, 571102, 570502) in the presence of Immunocult CD3/CD28/CD2 Human T cell Activator (StemCell Technologies; 10970). High-quality total RNA was isolated from fibroblasts using TRIzol Reagent (Invitrogen; 15596026). Next, complementary DNA was prepared using the SMART-Seq v4 Ultra Low Input RNA Kit for Sequencing (Takara). Library construction was performed using the NexteraXT DNA sample preparation kit (Illumina) using half the recommended volumes and reagents. Dual-index, single-read sequencing of pooled libraries was run on a HiSeq2500 sequencer (Illumina) with 58-base reads and a target depth of 5 million reads per sample. Base-calling and demultiplexing were performed automatically on BaseSpace (Illumina) to generate FASTQ files. The FASTQ files were processed to remove reads of zero length (fastq_trimmer v.1.0.0), remove adapter sequences (fastqmcf tool v.1.1.2), and perform quality trimming from both ends until a minimum base quality ≥ 30 (FASTQ quality trimmer tool v.1.0.0). Short read bulk RNA sequencing data were aligned to the human reference genome (build hg38) with TopHat (v.1.4.0) and read counts per Ensembl gene ID were quantified with htseq-count (v.0.4.1).^28^^,^^29^ Quality metrics for the FASTQ and BAM/SAM files were generated with FastQC (v.0.11.3) and Picard (v.1.128). Processing of FASTQ and BAM/SAM files was executed on the Galaxy workflow platform of Globus genomics. Statistical analysis of gene expression was assessed in the R environment (v.4.4.2; R Foundation for Statistical Computing). All samples passed a set of pre-established quality control criteria: total number of fastq reads >1 × 10^6^; mapped reads > 70%; median coefficient of variation of coverage <0.85. Randomization was not applicable as no treatment or intervention groups were included in the study. Blinding was not applicable as no treatment groups were compared.

### Additional bioinformatic analyses

Differential expression and pathway enrichment analyses were performed in R. To assess differential expression, the limma package (v.3.58.1) was used.^30^ The limma voomWithQuality weights transformation of TMM normalized counts was used to fit a linear model of gene expression as a function of recombinant cytokine treatment followed by empirical bayes smoothing of standard errors. A log2 expression fold change of at least 1 in magnitude and an false discovery rate of <0.05 were used as cutoffs to define differentially expressed genes. Overlap of differentially expressed genes and published fibrosis signatures was assessed using a hypergeometric test. Pathway enrichment analysis was performed using the enrichR package (v.3.2)^31^ with the Reactome 2022 database^32^ to query pathways associated with cytokine-induced genes.

Cytokine-dependent regulation of fibroblast gene expression using public data^33^ included all available T cell–stimulated samples (n = 69) of a distinct fibroblast donor. Measured cytokines were GM-CSF, IFN-γ, IL-2, IL-4, IL-5, IL-6, IL-9, IL-10, IL-13, IL-17a, IL-17f, IL-22, and TNF-α. Nonzero cytokine values were log10 transformed prior to analysis. For multivariable modeling, log2-transformed gene expression was tested as a function of log10-transformed cytokine concentrations using linear regression. Regression coefficient (β) represents the change in gene expression associated with a 10-fold increase in IFN-γ concentration, with all other cytokines included in the model.

### Statistics

To determine appropriate sample sizes a power analysis based on means and standard deviations from our published^10^ and preliminary immunophenotyping studies with PBMCs from SSc patients and healthy subjects was performed. To find an effect size of ∼50%, α = 0.05, power = 0.8, comparing expression of surface markers or transcription factors in defined cell subsets by flow cytometry, a sample size of n = 17 to 22 was calculated.

Statistical analyses were performed using Prism version 9.2.0.

Specific statistical analyses that were used for data in each figure are noted in the legends (i.e. Kruskal-Wallis test with Dunn’s test for multiple comparisons, 1-way analysis of variance followed by post hoc Tukey’s test for multiple comparisons, 2-way analysis of variance followed by post hoc Tukey’s test for multiple comparisons, and Student’s *t* test). Statistical significance was determined based on *P* ≤ 0.05. For all figures, data are shown as mean ± SEM or mean ± SD as indicated.

### Study approval

All samples and clinical data were collected upon written informed consent under a protocol approved by the Institutional Review Board at Boston University. Additional samples were obtained upon written informed consent at Virginia Mason Franciscan Health (Seattle, WA, USA), according to protocols approved by the Institutional Review Board of Benaroya Research Institute (Seattle, WA, USA). Cohort details for additional samples were reported previously.^33^

## Results

### Significant phenotypic heterogeneity within T cell lineages defined by transcription factor expression in human peripheral blood

We designed a multidimensional flow cytometry panel comprising of 20 markers to perform a comprehensive analysis of T cells expressing lineage-defining transcription factors in combination with markers of activation and dysfunction in T cells, including T-bet, GATA-3, RORγt, Bcl-6, Foxp3, TOX, PD-1, TIGIT, and CD226 (Table S1A). To validate that the intracellular markers faithfully identified distinct T cell subsets, we utilized opt-SNE analysis,^27^ generating heatmaps illustrating the expression levels of these markers (Fig. 1A–C). Canonical lineage-defining transcription factors showed expected expression patterns within CD4^+^, CD8^+^, and CD4^−^CD8^−^ T cell populations: for example, T-bet, GATA-3, and RORγt were present in all 3 T cell populations, while Foxp3 expression was confined to CD4^+^ and CD4^−^CD8^−^ T cells. Among CD4^+^ T cells, the majority of transcription factors were coexpressed with the CD45RO memory marker, confirming that they represented subsets of antigen-experienced T cells. In CD8^+^ and CD4^−^CD8^−^ T cells, some transcription factor–expressing subsets were detected within CD45RO^−^ T cells. These CD45RO^−^ subsets are likely part of the naïve T cell population or may be terminally differentiated effector memory T cells that re-express CD45RA.^34^ The latter designation cannot be made definitively though because CD45RA expression was not measured due to panel size limitations. Coexpression of most lineage-defining transcription factors was minimal, confirming the identification of discrete subsets (Fig. 1A–C). Thus, we have developed a unique flow cytometry panel that combines staining for an extensive number of intracellular proteins with viability, proliferation, and functional markers, allowing to faithfully and simultaneously identify all major functional T cell subsets in human blood and assess their biological state.

**Figure 1:**
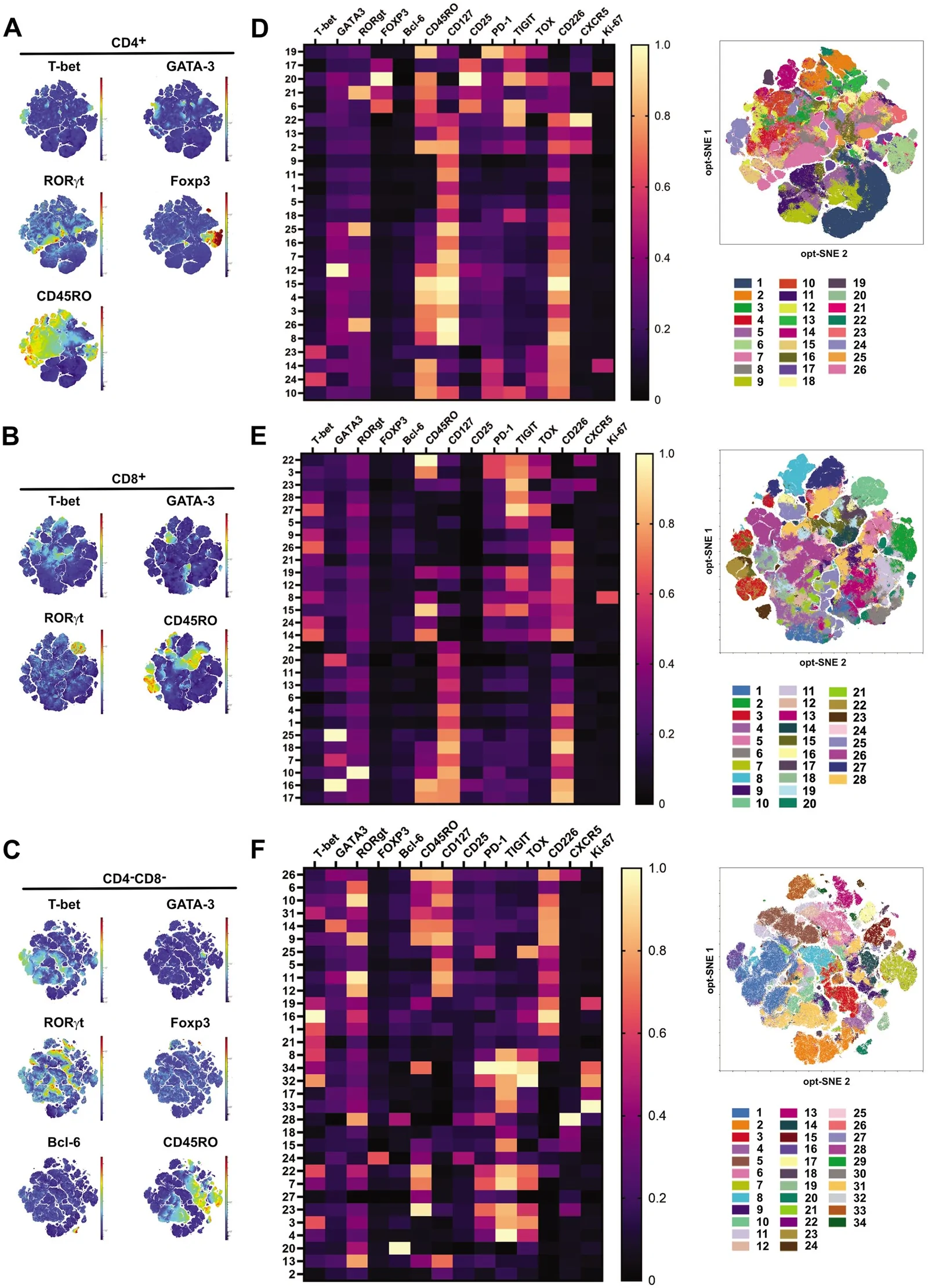
Identification of T cell subsets in human peripheral blood based on lineage-defining transcription factors reveals distinct functional phenotypes within canonical subsets. Peripheral blood samples of healthy subjects and dcSSc, lcSSc, and SLE patients were analyzed with a flow cytometry panel for full-spectrum flow cytometry (Table S1). Data were analyzed using opt-SNE algorithm and PhenoGraph clustering. Transcription factor expression patterns are shown as heatmaps overlaid on opt-SNE plots of live single CD3^+^ and (A) CD4^+^ T cells, (B) CD8^+^ T cells, and (C) CD4^−^CD8^−^ T cells. Normalized expression levels of intracellular and surface proteins in PhenoGraph clusters are shown in heatmaps for (D) CD4^+^ T cells, (E) CD8^+^ T cells, and (F) CD4^−^CD8^−^ T cells. Numbers represent the different, hierarchically arranged clusters. Values for every marker are normalized to minimum and maximum values in each column across CD4^+^, CD8^+^, and CD4^−^CD8^−^ T cell data for comparable analysis. Data include all samples concatenated.

To generate a detailed map of the phenotypical and functional diversity within T cell subsets, we employed the PhenoGraph algorithm, which identified 26 distinct clusters in CD4^+^, 28 clusters in CD8^+^, and 34 clusters in CD4^−^CD8^−^ T cells from all concatenated human peripheral blood samples (Fig. 1D–F). To identify the markers that defined each cluster, median fluorescence intensities within the PhenoGraph-generated clusters of CD4^+^, CD8^+^, and CD4^−^CD8^−^ T cells were plotted on hierarchically arranged heatmaps (Fig. 1D–F). Each marker expression was normalized across CD4^+^, CD8^+^, and CD4^−^CD8^−^ T cell populations to be able to compare differences in expression between these 3 T cell subsets. The combination of transcription factors and functional surface receptors detected with our panel revealed previously unknown phenotypes within canonical T cell subsets in human peripheral blood, allowing to compare the frequencies of these subsets in healthy and diseased individuals. Some noted features included that CD8^+^ and CD4^−^CD8^−^ T cells contained more individual clusters defined by T-bet expression than CD4^+^ T cells, while, as expected, Foxp3^+^ T cells were largely confined to CD4^+^ T cells. Interestingly, large areas within the CD45RO^+^ T cell region did not show expression of a canonical transcription factor, suggesting the prevalence of uncommitted, reverted or undefined cell populations. Many clusters expressed the co-SR CD226, while its competing co-IR TIGIT, which binds to the same ligands, showed more limited cluster distribution. GATA-3 was only highly expressed in one cluster in CD4^+^ and CD4^−^CD8^−^ T cells but multiple among CD8^+^ T cells. CD4^−^CD8^−^ T cells contained several clusters positive for RORγt, while CD4^+^ and CD8^+^ T cells showed less diversity in RORγt^+^ cells. This unbiased clustering analysis hence documented the T cell landscape in human peripheral blood and revealed previously unknown details of T cell subset composition.

### Features of a compromised type 1 T cell compartment dominate the altered T cell landscape in SSc and SLE patients

Next, we leveraged our dataset to assess differences in cluster frequencies between healthy subjects, SSc patients, and SLE patients. For this analysis, we used a carefully characterized patient cohort representing SSc disease heterogeneity. SSc and SLE cohort characteristics are shown in Table 1, demonstrating that healthy and SSc cohorts were largely age and sex matched, while SLE patients tended to be younger, consistent with typically earlier disease onset. The SSc population consisted of lcSSc (n = 21) and dcSSc (n = 22) patients with and without SSc interstitial lung disease (SSc-ILD) and pulmonary arterial hypertension. It is also important to note that patients that were not taking immunosuppressive drugs and patients that were treated with these drugs were both represented, allowing to evaluate the impact of drug treatment on the T cell landscape.

**Table 1:** Clinical information of healthy subjects and SSc and SLE patients.

Characteristic	Healthy subjects (n = 17)	dcSSc (n = 22)	lcSSc (n = 21)	SLE (n = 15)
**Male/female**	4/13	5/17	3/18	2/13
**Age, yr**	52 (30–69)	53 (39–68)	60 (25–87)	40 (26–71)
**Disease duration**	N/A	4 yr (1 mo-17 yr)	9 yr (1–24 yr)^a^	4.5 yr (0.5–12 yr)
**Immunosuppressants, no/yes**	N/A	13/9	13/8	11/4
**Hispanic White/Black/White/Asian**	1/2/11/0	0/1/20/1	0/5/16/0	3/9/1/2
**Modified Rodnan skin score**	N/A	9 (0–31)^b^	3 (0–14)^b^	N/A
**ILD**	N/A	9	12	N/A
**PAH**	N/A	1	6	N/A

Values are n or median (interquartile range).

Abbreviations: N/A, not applicable; PAH, pulmonary arterial hypertension.

a Disease duration was not available for 3 lcSSc patients.

b No modified Rodnan skin score was reported for 1 dcSSc patient and 3 lcSSc patients.

Generally, we observed a shift toward increased presence of CD45RO^+^ clusters in SSc and SLE patients, which indicates a more “aged” and/or active immune system as has been described for systemic autoimmune diseases (Fig. S1).^35^ Next, to evaluate the paradigm that fibrosis in SSc is driven by type 2 immune cells,^36^^,^^37^ we analyzed the representation of T cell subsets expressing the canonical type 2 transcription factor GATA-3. Surprisingly, we found that within CD4^+^ T cells, the frequencies of the CD45RO^+^ GATA-3–expressing clusters 12 (GATA-3^high^) and 15 (GATA-3^low^) were not significantly different in SSc patients versus healthy subjects (Fig. 2A). In SLE patients, GATA-3–expressing cells were significantly reduced compared with both healthy subjects and dcSSc patients. Similarly, within CD8^+^ T cells, GATA-3–expressing clusters were not altered in SSc patients but were reduced in SLE patients (Fig. 2B). The main GATA-3^+^ subset within CD4^−^CD8^−^ T cells showed no differences between the cohorts (data not shown). Hence, SSc patients did not show an increased bias toward a type 2 immune landscape in peripheral blood, as concluded from a lack of expansion of GATA-3^+^ subsets. This appears to contrast with reports that show an increase in Th2/Tc2 cytokines in SSc patients compared with healthy subjects.^38–40^ Hence, it is feasible that GATA-3^+^ T cells are not more numerous in SSc but exhibit a lower threshold for cytokine production. On the other hand, our data revealed that a significant reduction in multiple GATA-3–expressing T cell subsets is a disease-specific feature of SLE.

**Figure 2:**
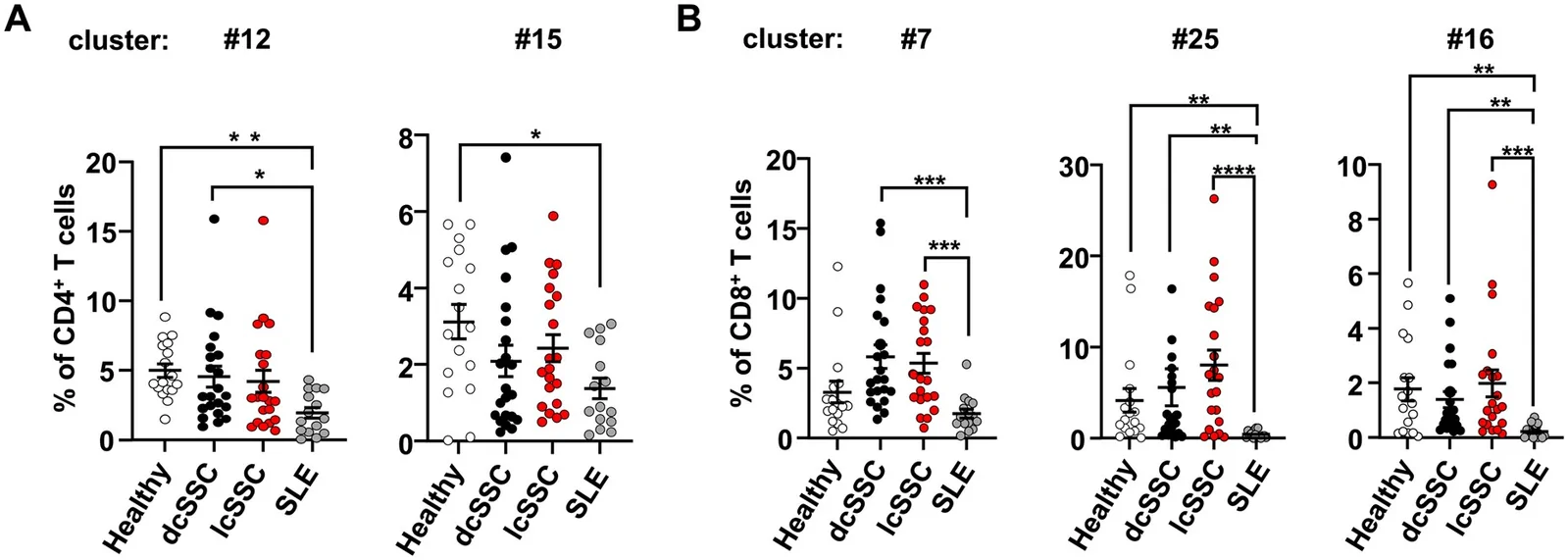
SSc patients do not show major changes in GATA-3–expressing T cells. Frequencies of GATA-3–expressing clusters were determined using markers that characterized the cluster as identified in heatmaps from Figure 1D and E, using OMIQ software. Cell frequencies of indicated clusters within parental populations of (A) CD4^+^ and (B) CD8^+^ T cells in healthy subjects (n = 17, open circles) and dcSSc (n = 22, black circles), lcSSc (n = 21, red circles), and SLE (n = 15, gray circles) patients are shown. Graphs show percentages of gated cells of indicated clusters with mean and SEM. Each symbol represents a blood sample from an individual donor. Data were statistically analyzed with the Kruskal-Wallis test followed by Dunn’s multiple comparisons test. *P < 0.05, **P < 0.01, ***P < 0.001, ****P < 0.0001.

To assess prevalence of type 1 immunity, we analyzed T-bet–expressing clusters. Within CD4^+^ T cells, the two main T-bet^+^ clusters, 23 and 24, which were distinguished by expression of CD45RO and typically represented <10% each of CD4^+^ T cells, were increased in SLE patients but not in SSc patients, compared with healthy subjects (Fig. 3A). T-bet directs Th1 lineage commitment, and T-bet–expressing CD4^+^ T cells are known to be increased in SLE patients and may have a pathophysiological role, underscoring that our findings confirm previous studies.^41^ Within CD8^+^ T cells, many T-bet–expressing clusters were identified, with 14, 26, and 27 representing the most cells. The CD45RO^+^ T-bet^+^ cluster 14 that expressed CD226, TIGIT and TOX, and the CD45RO^−^ T-bet^+^ cluster 26, characterized by CD226, TOX and PD-1 expression, showed no difference in SSc patients and a minor increase in SLE patients, not reaching statistical significance (data not shown). Strikingly, cluster 27, composed of T-bet^+^ CD45RO^−^ CD127^−^CD226^−^TOX^high^TIGIT^high^ CD8^+^ T cells, was substantially reduced in SSc and SLE patients compared with healthy subjects (Fig. 3B). This cluster represented the largest number of T-bet–expressing CD8^+^ T cells in healthy subjects, and the high levels of TOX and TIGIT, along with the absence of co-SR CD226, suggests this is a dysfunctional population of Tc1 cells. Similarly, in CD4^−^CD8^−^ T cells, cluster 16, which expressed high levels of T-bet, was significantly reduced in all 3 disease types (dcSSc, lcSSc, and SLE) compared with healthy subjects (Fig. 3C). Cluster 31 was also reduced in SLE and showed a similar trend in SSc. Staining with anti-Vδ2 T cell receptor antibodies revealed that these clusters belonged to the Vδ2^+^ subset of γδ T cells, the most ubiquitous γδ T cell population in human blood.^42^ Cluster 32, on the other hand, representing Vδ1^+^ γδ T cells, was increased in SLE and exhibited a highly activated phenotype, characterized by coexpression of PD-1, TIGIT, TOX, and the proliferation marker Ki-67 (Fig. 3C). Hence, we conclude that SSc and SLE patients share an autoimmune signature characterized by an altered type 1 immune compartment compared with healthy subjects. This signature is not defined by a broad and indiscriminate reduction of T-bet–expressing T cells but by shared deficiencies in specific subsets. Moreover, further support for a dysfunctional type 1 immune response in SSc and SLE patients came from the finding that frequencies of Foxp3^+^ Tregs expressing high levels of TIGIT (cluster 6), a subset that has been associated with preferential suppression of Th1 but not Th2 cells, were increased (Fig. 3D).^20^^,^^43^ A second TIGIT^+^ Treg cluster, 20, also showed a trend to be increased in dcSSc patients but not in lcSSc and SLE patients. The main difference between clusters 6 and 20 is that cluster 20 expressed Ki-67 and TOX, indicating a highly activated, proliferating population of TIGIT^+^ Tregs (Fig. 3D). Treg clusters (17 and 21) that expressed low levels of TIGIT did not show a difference between healthy subjects and patients (data not shown). In conclusion, analysis of T cell subsets expressing GATA-3, T-bet, or Foxp3 in human peripheral blood revealed that changes are not uniform across all cells expressing these lineage-defining transcription factors. Instead, specific, previously undefined subsets within these populations are altered in frequencies in a disease-specific manner, and these changes are indicative of a dysregulated type 1 immune response in SSc and SLE.

**Figure 3:**
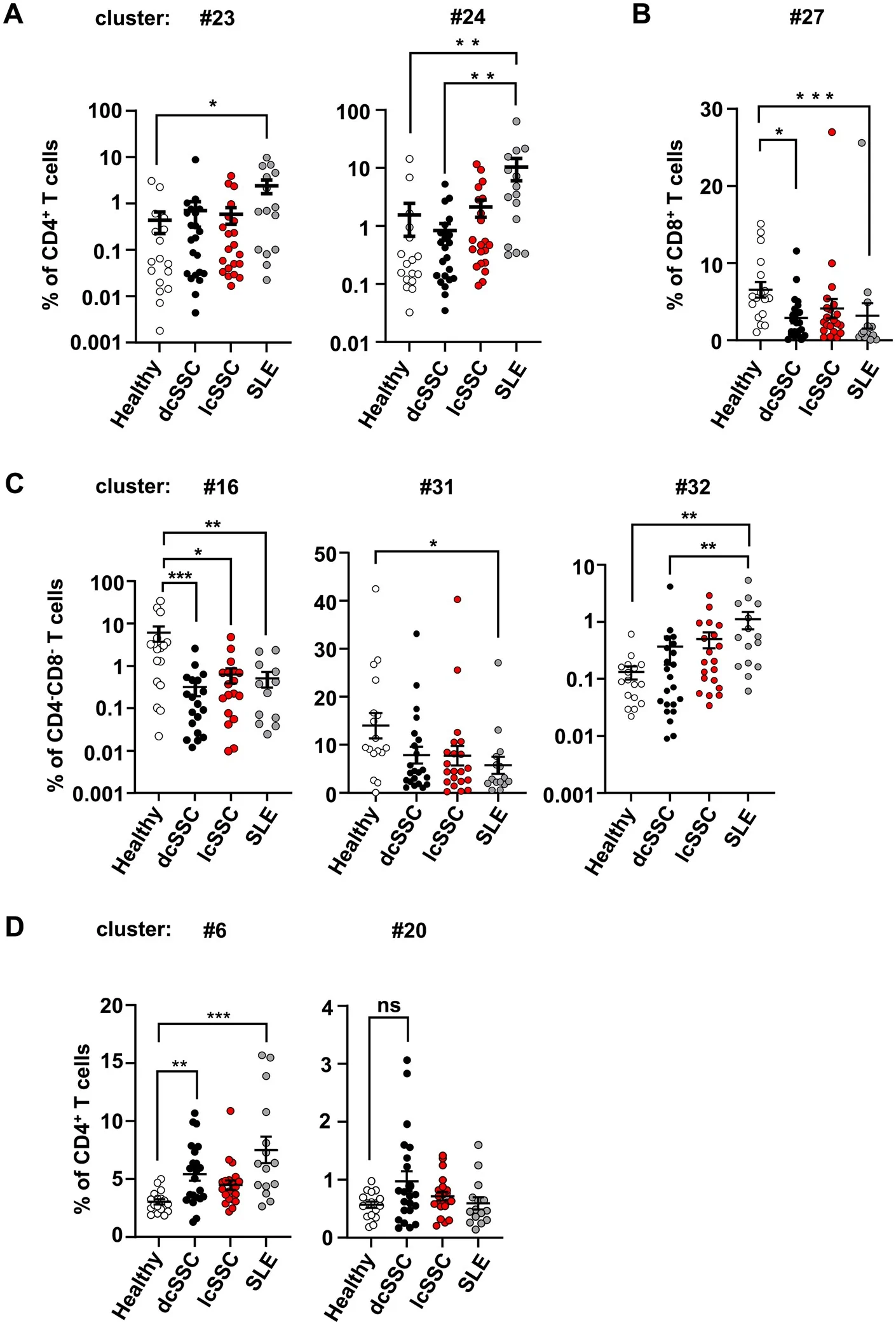
SSc and SLE patients exhibit features of reduced type 1 immunity. Frequencies of T-bet– and Foxp3-expressing clusters were determined using markers that characterized the cluster as identified in heatmaps from Figure 1D–F, using OMIQ software. Cell frequencies of indicated T-bet–expressing clusters within parental populations of (A) CD4^+^, (B) CD8^+^, and (C) CD4^−^CD8^−^ T cells, and cell frequencies of (D) indicated Foxp3-expressing clusters within CD4^+^ T cells are shown for healthy subjects (n = 17, open circles) and dcSSc (n = 22, black circles), lcSSc (n = 21, red circles), and SLE (n = 15, gray circles) patients. Graphs show percentages of gated cells of indicated clusters with mean and SEM. Each symbol represents a blood sample from an individual donor. Data were statistically analyzed with the Kruskal-Wallis test followed by Dunn’s multiple comparisons test. *P < 0.05, **P < 0.01, ***P < 0.001. ns, not significant.

### Select type 17 T cell subsets are sensitive to immunosuppressive drugs in SSc patients

Next, we analyzed the composition of RORγt-expressing CD4^+^, CD8^+^, and CD4^−^CD8^−^ T cells in our patient cohorts. In CD4^+^ T cells, RORγt expression defines the Th17 lineage.^44^ Th17 cells are expanded in peripheral blood of SSc patients, but their role in the disease remains poorly defined^7^^,^^45–47^; in SLE, Th17 cells are thought to exacerbate the disease.^48^^,^^49^ In our study, we found that 3 CD4^+^ clusters expressed high levels of RORγt: cluster 21 coexpressed Foxp3 and has been discussed previously, clusters 25 and 26 were distinguished by CD45RO expression. CD45RO^−^ RORγt-expressing T cells constituted on average < 2% of CD4^+^ T cells and were increased in dcSSc and SLE patients compared with lcSSc patients, which had very low levels of these cells (Fig. 4A). Interestingly, separating SSc patients based on immunosuppressive drug treatment revealed that the higher frequencies of RORγt^+^ T cells in dcSSc were driven by patients that were not taking immunosuppressive drugs (Fig. 4A). Treatment with immunosuppressants reduced RORγt^+^ T cells back to levels seen in healthy subjects and lcSSc patients, showing specific expansion of this population in dcSSc patients is sensitive to immunomodulation. Cluster 26, representing CD45RO^+^ effector/memory RORγt^+^ T cells, showed only minor differences between patient cohorts (data not shown).

**Figure 4:**
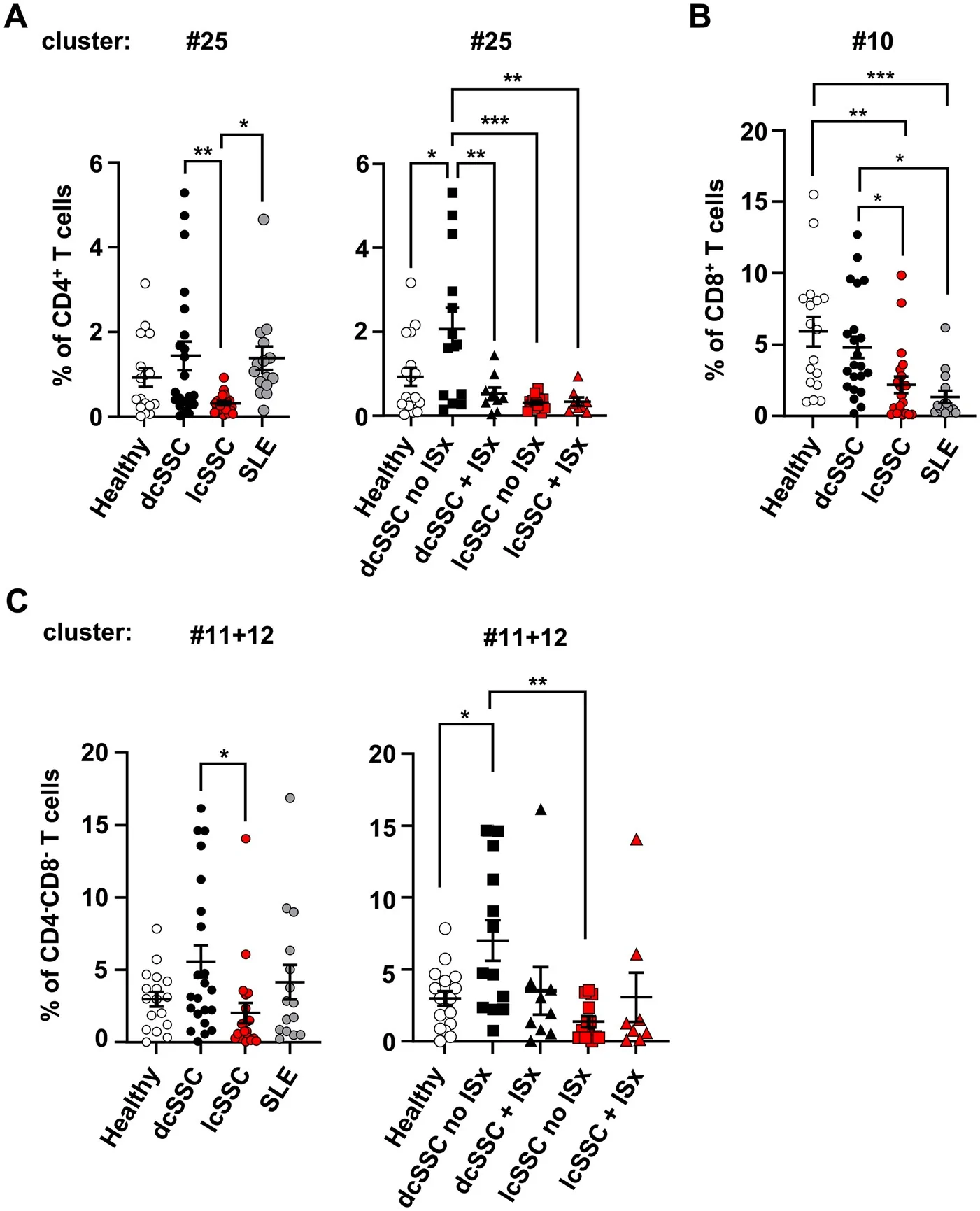
RORγt-expressing T cell clusters show disease-specific changes in frequency and are sensitive to immunomodulation in dcSSc patients. Frequencies of RORγt-expressing clusters were determined using markers that characterized the cluster as identified in heatmaps from Fig. 1D–F, using OMIQ software. Cell frequencies of indicated RORγT-expressing clusters within parental populations of (A) CD4^+^, (B) CD8^+^, and (C) CD4^−^CD8^−^ T cells, in healthy subjects (n = 17, open circles) and dcSSc (n = 22, black circles), lcSSc (n = 21, red circles), and SLE (n = 15, gray circles) patients are shown. In the right panels of (A) and (B) data from dcSSc and lcSSc patients separated based on immunosuppressant treatment (ISx) (no ISx, squares; with ISx, triangles) are shown. Graphs show percentages of gated cells of indicated clusters with mean and SEM. Each symbol represents a blood sample from an individual donor. Data were statistically analyzed with 1-way analysis of variance followed by Tukey’s multiple comparisons test. *P < 0.05, **P < 0.01, ***P < 0.001.

In CD8^+^ T cells, RORγt expression defines the IL-17-producing subset (Tc17), which is a relatively recently discovered population of cells that has been shown to be involved in autoimmune, infection, and cancer settings.^50^ We observed a significant reduction in Tc17 cells in lcSSc patients, compared with healthy subjects and dcSSc patients (cluster 10) (Fig. 4B). Our data align with another recent report showing a reduction in Tc17 cells in SSc patients, underscoring the validity of this finding and supporting a potentially important functional role for these cells in SSc.^39^ Surprisingly, in contrast to previous reports in the literature showing that the proportion of Tc17 cells was increased in blood from SLE patients,^48^ we observed a reduction of Tc17 cells in SLE patients as well.

Within the CD4^−^CD8^−^ T cell population many RORγt^+^ clusters were detected, which varied in CD45RO, CD127, and CD226 expression (data not shown). However, most of these clusters did not differ in frequency between the cohorts. Clusters 11 and 12 were combined due to their similarity in RORγt^high^ and GATA-3^low^ expression and were significantly increased in dcSSc patients compared with lcSSc patients (Fig. 4C). This increase was sensitive to immunosuppressant treatment (Fig. 4C). Notably, these clusters did not coexpress any of the γδ T cell markers, and hence they presumably belong to a subset of iNKT cells that is designated as NKT17.^51^ Interestingly, sensitivity to immunosuppressant drugs appeared to be mainly a feature of RORγt-expressing T cell subsets as analysis of the other transcription factor–expressing T cell subsets did not show a significant difference with and without treatment.

### Additional aberrant features of the T cell landscape in SSc and SLE

Several additional observations of interest were made, including a significant reduction of a cluster (2) of CXCR5-expressing CD45RO^+^ CD4^+^ T cells in dcSSc and SLE patients, while lcSSc patients had normal levels of these cells, indicating a possible disease-specific role in dcSSc and SLE (Fig. S2A). This cluster likely represents a population of blood memory Tfh cells, which often lack Bcl-6 expression.^52^ Elevated levels of Tfh cells have been found in SSc patients and correlated with lung disease severity.^53^^,^^54^ Within the CD4^−^CD8^−^ T cell population, a subset that expressed Bcl-6 in combination with a detectable level of RORγt (cluster 20) was significantly increased in SLE patients, and perhaps marginally in lcSSc as well (Fig. S2B). This subset did not express Vδ1 or Vδ2, suggesting that these are likely iNKT cells. Although the existence of follicular helper iNKT cells that express Bcl-6,^55^^,^^56^ as well as RORγt^+^ iNKT cells,^57^^,^^58^ is well established, there are no available reports of iNKT subsets that coexpresses both Bcl-6 and RORγt. Finally, we analyzed differences in clusters identified in Figure 1D–F that were not defined by transcription factor expression but showed features of interest, including sensitivity of some clusters to immunosuppressant treatment; notable findings are shown in Fig. S3.

### Reduced production of the antifibrotic cytokine IFN-γ by circulating T cells from SSc patients

Our finding that SSc patients showed irregularities in T-bet–expressing T cell subsets and Tregs prompted the question whether this was associated with deficiencies in production of the signature type 1 cytokine IFN-γ. Reduced IFN-γ production may have a profound impact on the control of fibrosis as it has been reported this cytokine has antifibrotic properties.^18^^,^^19^^,^^59–61^ To assess IFN-γ production in SSc donor T cells, we used blood samples curated from a cohort of SSc subjects at the Benaroya Research Institute who were not treated with immune-modifying drugs.^33^ Clinical characteristics of this cohort have been previously published.^33^ Pan-T cells from these SSc patients (n = 17) and sex- and age-matched healthy subjects (n = 15) were isolated and stimulated with PMA/ionomycin followed by intracellular cytokine staining. We observed no major differences in total T cell or memory population frequencies between study groups (Fig. S4A–D). Consistent with a defect in type 1 immunity, we found that memory CD4^+^ and CD8^+^ T cells from SSc subjects showed a marked reduction in IFN-γ production (Fig. 5A, B). We also observed a small but significant increase in IL-13 production by memory CD8^+^ T cells, but not by CD4^+^ T cells (Fig. 5A, C), consistent with previous studies.^1^^,^^9^

**Figure 5:**
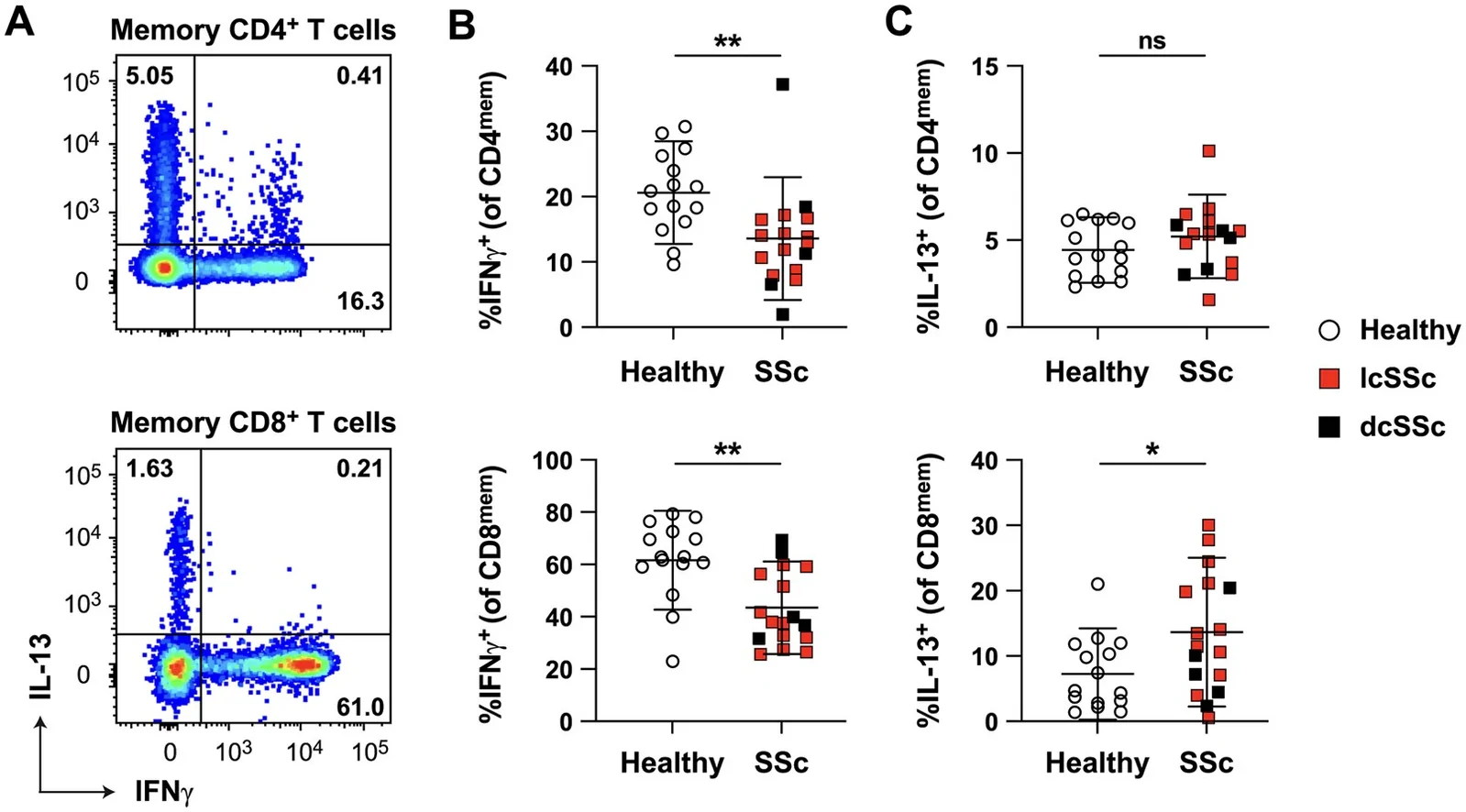
Peripheral blood T cells from SSc patients show reduced production of the antifibrotic cytokine IFN-γ. Intracellular cytokine analysis of memory CD4^+^ (top) and CD8^+^ (bottom) T cells from healthy subjects (n = 15, open circles) and SSc (n = 17, lcSSc: red squares; dcSSc: black squares) donors following PMA/ionomycin stimulation. (A) Representative plots of indicated memory T cells showing IFN-γ and IL-13 expression. (B, C) Frequencies of cytokine-producing memory T cells across subject groups showing (B) IFN-γ^+^ and (C) IL-13^+^ cells as a percentage of CD4^+^ or CD8^+^ memory T cell subsets. Each point represents an independent biological replicate. Statistical significance: *P < 0.05, **P < 0.01 (unpaired *t* test or Mann-Whitney *U* test based on Shapiro-Wilk distribution testing). Error bars show mean ± SD. ns, not significant.

To determine whether IFN-γ production by T cells can oppose fibrotic gene programs, consistent with its reported role in connective tissue disorders,^19^^,^^59^ we compared transcriptional responses of healthy human fibroblasts exposed for 24 h to recombinant IFN-γ, IL-13, or IL-17a. Dimensionality reduction and differential gene expression analyses revealed that IFN-γ had the greatest impact on fibroblast gene expression, with more subtle effects in response to IL-13 or IL-17a (Fig. 6A; Fig. S5A–C). We next assessed overlap with public transcriptomic signatures shared across fibrotic diseases, including SSc,^62^^,^^63^ and found a significant enrichment of fibrosis-associated gene sets among the cytokine-responsive genes in dermal fibroblasts (Fig. 6B). IFN-γ strongly promoted canonical IFN-responsive pathways, while suppressing matrix production and regulatory programs that were induced by IL-13 (Fig. 6B, C; Fig. S5A, B). Consistent with this, analysis of curated IFN-responsive and fibrosis-associated genes revealed opposing effects of IFN-γ and IL-13 in fibroblasts (Fig. 6D, E). IFN-γ selectively downregulated the expression of multiple collagen family genes and antagonized common stem-like, wound healing, and myofibroblast-related programs (Fig. 6E).

**Figure 6:**
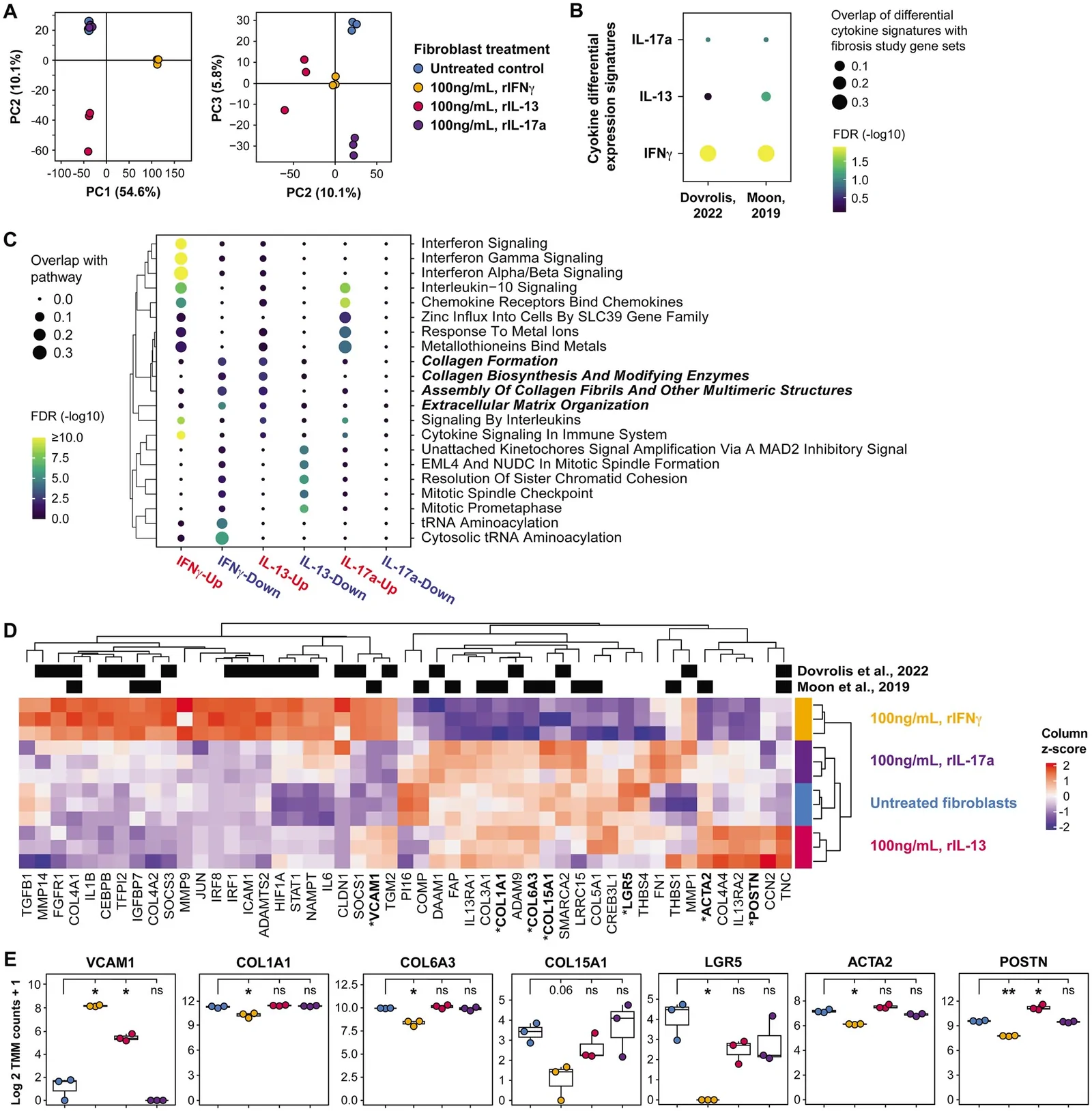
Cytokine-mediated transcriptional perturbation of fibroblasts reveals an antifibrotic effect of IFN-γ. Transcriptional analysis of healthy donor fibroblasts treated with IFN-γ, IL-13, or IL-17a (100 ng/mL) versus untreated controls. (A) Principal component (PC) analysis of fibroblast gene expression following cytokine treatment showing PC1, PC2, and PC3. (B) Dot plot showing the overlap of cytokine-induced fibroblast gene signatures from panel A with transcriptional profiles taken from indicated published fibrosis datasets, Dovrolis et al.^63^ or Moon et al.^62^ (C) Enrichment of Reactome pathways across cytokine-treated fibroblasts, with the direction and strength of enrichment indicated (cytokine effect on pathway, red = up; blue = down). (D) Heatmap shows IFN-stimulated and fibrosis-associated transcripts regulated by cytokines (*z* score normalized); genes overlapping with public datasets in panel B are marked. Bolded genes marked with an asterisk are plotted in panel E. (E) Log2-transformed expression of select genes in fibroblasts treated with recombinant IFN-γ, IL-13, or IL-17a. Each point represents an independent biological replicate. Statistical significance: *P < 0.05, **P < 0.01 (unpaired *t* test or Mann-Whitney *U* test based on Shapiro-Wilk distribution testing). Error bars show mean ± SD. FDR, false discovery rate; ns, not significant.

As an orthogonal approach, we analyzed a public dataset of T cell supernatant-driven fibroblast responses in healthy donors.^33^ Increased T cell production of IFN-γ corresponded with reduced expression of SSc-related genes, including the myofibroblast marker POSTN and the matrix gene COL6A3 (Fig. S5D). Multivariable modeling accounting for 13 T cell cytokines demonstrated an independent, negative association between IFN-γ concentration and fibroblast expression of COL6A3, LGR5, and POSTN, consistent with effects observed following recombinant cytokine stimulation (Fig. S5E). Together, these data support a model in which IFN signaling antagonizes fibrotic gene programs and suggest that the defective type 1 immunity in SSc may contribute to a dysregulated wound healing response.

## Discussion

Analysis of lineage-defining transcription factors in individual T cells offers insights in the functional capabilities of these cells without altering their phenotype through in vitro restimulation. To understand differences in functional T cell subsets in peripheral blood from patients with the systemic autoimmune diseases SSc and SLE, we designed a multidimensional spectral flow cytometry panel including the lineage-defining transcription factors T-bet, GATA-3, RORγt, Bcl-6, and Foxp3, combined with phenotypical and functional receptors and additional intracellular molecules. We hypothesized that this analysis would reveal previously unknown changes in frequency and functionality of pro- and antifibrotic T cell subsets in SSc patients, advancing our understanding of this disease. Automated clustering analysis of the datasets generated from cohorts of lcSSc, dcSSc, and SLE patients, and healthy subjects, showed undescribed granularity in T cell subsets defined by transcription factor expression. Previously unknown features of the circulating T cell landscape emerged, distinguishing dcSSc, lcSSc, and SLE patients and healthy subjects. Although SSc has been described as a disease driven by T cell–derived type 2 cytokines that exert profibrotic activities on fibroblasts,^36^ we surprisingly did not find a clear difference in the frequencies of GATA-3–expressing T cells. Rather, we found a prominent decrease in some subsets of T-bet–expressing cells within the CD8^+^ and CD4^−^CD8^−^ γδ T cell populations. This finding, together with our separate observation of reduced IFN-γ production in SSc T cells, indicates a defective type 1 immune compartment that may play a role in disease etiology through a mechanism that remains to be defined.

Decreased circulating CD8^+^ T cell numbers and dysfunctional CD8^+^ T cells have been shown in SSc.^40^^,^^64^ Fuschiotti et al.^9^ demonstrated that CD8^+^ T cells from SSc patients contain a population of IL-13–producing cells with profibrotic activity. Mechanistically, this was associated with cytosolic sequestering of T-bet, a known promoter of type 1 cytokine production and consequently an inhibitor of profibrotic type 2 cytokines.^38^ Recently, Padilla et al.^65^ showed increased CD8^+^ tissue resident memory T cells in lungs from SSc-ILD patients, but their functional properties remain unclear. γδ T cells are important sources of IFN-γ and TNF-α and have been proposed to play a role in autoimmune rheumatic diseases, including SSc.^66^ Circulating γδ T cells numbers were shown to be lower in peripheral blood of SSc patients but were activated and stimulated procollagen gene expression in fibroblasts^67^ and interacted with endothelial cells.^68^ Moreover, γδ T cells appeared to accumulate in SSc skin.^69^ Our analysis of CD4^−^CD8^−^ γδ T cells provides new information about the specific subpopulations of γδ T cells that are reduced in SSc (i.e. the ones expressing T-bet).^70^^,^^71^ Some authors have described γδ T cells with antifibrotic properties, and this may be related to their strong capacity for producing IFN-γ and TNF-α, cytokines that kill aberrant fibroblasts.^61^^,^^67^ Moreover, studies in mice have demonstrated that T-bet deficiency exacerbated lung and skin fibrosis in multiple models.^15–17^ The 3 separate components of our study, namely (i) reductions in circulating CD8^+^ and γδ T cells that express T-bet, (ii) reduced capacity to produce IFN-γ in SSc T cells, and (iii) suppression of profibrotic gene expression by IFN-γ in fibroblasts, form the initial framework for a model in which deficient type 1 immunity contributes to fibrosis. To overcome the limitations of our study with in vitro fibroblast cultures, direct evidence of temporal and spatial links between the deficient T cell subsets and fibroblast activation within intact tissues will be needed to validate this mechanism in SSc.

Type 1 immunity in dcSSc patients may be further restrained by the increased presence of a specialized Foxp3^+^ Treg subpopulation expressing TIGIT. TIGIT-expressing Foxp3^+^ Tregs have been associated with selective suppression of Th1 and Th17 responses by increasing IL-10 production in dendritic cells,^20^^,^^43^ further underscoring the presence of an immune landscape characterized by compromised type 1 immune activity. Interestingly, a recent study showed that TIGIT engagement in Foxp3^+^ Tregs induces tissue repair functions through the secretion of the tissue growth factor amphiregulin, suggesting that increased presence of these cells may contribute to excessive tissue repair leading to fibrosis.^72^ It is important to note here that the TIGIT-expressing Treg clusters were increased in dcSSc patients but not in lcSSc patients. dcSSc and lcSSc patients differ in skin and organ involvement, as well as in autoantibody profile. These distinct phenotypes are likely caused by different underlying pathophysiological mechanisms. It will be of interest to investigate whether a distinct immunopathologic role of TIGIT^+^ Tregs in dcSSc vs lcSSc represents one mechanism that contributes to underlying differences in dcSSc and lcSSc disease presentation.

Existing literature on the balance of type 1/type 2/type 17 cells and their complex regulation by immune checkpoints has not led to an overall coherent concept of their role in SSc.^39^^,^^73^^,^^74^ This may be related to the heterogeneity of the studied patient cohorts, but also because the current mapping of altered T cell subsets is incomplete. Nevertheless, some common patterns have emerged, and our data provide new pieces that will advance the development of a concept to understand the role of immune dysregulation in SSc. Specifically, we propose a model in which the loss of T-bet–expressing subsets precedes, and therefore enables, the development of the profibrotic type 2 response. Future investigations to establish this timeline experimentally and determine the causes of the defective T-bet^+^ compartment in subjects at risk for SSc will be required to understand the role of type 1 immune dysregulation in SSc etiology. Additionally, altered Th17, Tc17, and Treg frequencies have been reported in SSc, but consensus on their role in inflammation and fibrosis is lacking.^39^^,^^73–76^ In the case of Tregs and type 17 cells, our findings, together with other recent studies,^39^^,^^72^^,^^76^^,^^77^ suggest that focusing on the functions of TIGIT^+^Fopx3^+^ Tregs and Tc17 cells specifically may mitigate some of the conflicts generated by studies based on broader cell populations.

Our findings also have translational implications. Although previous attempts to treat fibrotic conditions with IFN-γ were largely unsuccessful,^78^^,^^79^ our study serves as a resource for identifying candidate cell subsets that may be worth restoring in SSc patients for therapeutic benefit. In mouse models, agents that promote the IFN-γ/Th1 response and reduce Th2 immunity have been shown to protect against pulmonary fibrosis.^80^ Nevertheless, caution is warranted, as T cell function is often highly dependent on the tissue cytokine microenvironment, and IFN-γ–secreting populations have been associated with both antifibrotic and profibrotic activities in various liver diseases for example.^81^^,^^82^ In SSc, a recent study showed the presence of CD8^+^ T cells and inflammatory fibroblasts in the skin, with the latter cells exhibiting a type II IFN-induced gene signature,^83^ underscoring that IFN-γ function is likely dependent on disease stage and tissue location. Therefore, a limitation of our study is that phenotypically and functionally altered T cell subsets identified in the circulation cannot directly be assumed to be similarly altered in affected tissues. It is encouraging though that several recent studies demonstrate that novel T cell subsets discovered in the blood of SSc patients are present in skin or lung tissues and are associated with severity of SSc-ILD.^5^^,^^84–86^

For our study, we chose to extend our analysis to T cells from another systemic rheumatic disease, SLE, to reveal unique features of the SSc T cell landscape. In fact, we discovered that the severe reduction in specific type 1 T cell subsets was shared between these diseases. Interestingly however, the T cell landscape in SLE did not only share general features with SSc but also specific subset changes that differentiated lcSSc and dcSSc (e.g. in Tc17 cells and TIGIT^+^ Tregs). In addition, SLE contained other, disease-specific changes such that the overall T cell landscape in SLE clearly differed from SSc. It is important to recognize that our comparison between SSc and SLE patients is subject to limitations such as differences in age distribution and treatment exposure. Despite these challenges in study design, we propose that the altered balance and composition of the aberrant T cell subsets in each disease may lead to differential functional integration that underlies unique disease pathophysiology.

Finally, we acknowledge that immunosuppressive treatment represents a potential source of residual confounding effects and hence is a limitation of the study. Although we examined its impact through stratified analyses, the limited sample size constrained our ability to perform more fully adjusted modeling. Interestingly, using stratified analyses, we report the surprising finding that some T cell subsets are more prone to immune-modifying treatments than others. While we did not observe significant sensitivity of T-bet or GATA-3–expressing subsets to immunosuppression, RORγt-expressing subsets appear particularly sensitive to immune-modifying therapies and were restored to normal levels in patients treated with immunosuppressive drugs. Whether restoration of these specific subsets contributes to symptom alleviation and slowed disease progression remains unanswered but worth exploring. Together, our study provides a map to identify T cell subsets that are the most likely impactful targets of immune modulation in SSc and SLE, revealing novel candidates for T cell-based precision therapies to repair the aberrant immune environment underlying pathology.

## Supplementary Material

vkag288_Supplementary_Data

## Data Availability

All datasets generated and analyzed in this study are available from the corresponding authors on reasonable request. Frequency data for all the clusters identified in Figure 1D–F are available as Supplementary Material. All gene expression datasets generated in this study are available to the public in the Gene Expression Omnibus (GSE305982). Computer code generated and used for analysis in this study is available in github: https://github.com/BenaroyaResearch/VolfsonAlteredTCellLandscapeInSScAndSLE.
