# Supplementary material for: The altered T cell landscape in systemic sclerosis patients is characterized by dysfunctional type 1 immunity
Source: J Immunol. 2026 Nov 4;215(11):vkag288. doi: 10.1093/jimmun/vkag288 (PMC13735513; doi:10.1093/jimmun/vkag288)
Supplement: Supplementary file 1 [file vkag288_supplementary_data.zip › Supplementary Material Volfson-Sedletsky.pdf]

A

Specificity	Fluorophore	Vendor	Catalog #	Clone	Staining concentration
L/D	UV Zombie	Biolegend	423107	-	1:800
CD25	BUV395	BD	564034	2A3	1:50
CD127	BUV737	BD Biosciences	612795	HIL-7R-M21	1:50
PD-1	BV421	Biolegend	329920	EH12.2H7	1:50
CD3	BV510	Biolegend	317332	OKT3	1:100
TIGIT	BV605	Biolegend	372712	A15153G	1:50
T-bet	BV711	Biolegend	644820	4B10	1:50
CXCR5	BV750	Biolegend	356942	J252D4	1:25
CD226	BV786	BD Biosciences	742497	DX11	1:50
RORγt	AF488	BD Biosciences	563621	Q21-559	1:20
Ki-67	AF532	eBioscience/ ThermoFisher	58-569-882	SolA15	1:200
CD4	PerCP-e710	eBioscience/ ThermoFisher	50-112-9403	SK3	1:3200
FOXP3	PE	eBioscience/ ThermoFisher	12-4776-42	PCH101	1:100
Bcl-6	PE/Dazzle594	Biolegend	358510	7D1	1:40
GATA3	PE-Cy7	eBioscience/ ThermoFisher	25-9966-42	TWAJ	1:5
TOX	APC	Miltenyi	130-118-335	REA473	1:100
CD45RO	Alexa700	BD Biosciences	561136	UCHL1	1:50
CD8	APC-Fire/810	Biolegend	344764	SK1	1:100
Vδ1	APC-Vio770	Miltenyi	130-120-438	REA173	1:100
Vδ2	PerCP	Biolegend	331410	B6	1:50

B

Specificity	Fluorophore	Clone	Vendor	Catalog #	RRID #	Staining concentration (ug/mL)
Identification and cytokine staining of blood-derived T cells (Figure 5, Supplemental Figure 4)	-	-	-	-	-	-
CD3	BUV395	OKT3	Thermo Fisher Scientific	363-0037-42	RRID:AB_2925248	1
GM-CSF	BV421	BVD2-2C11	BD Biosciences	562930	RRID:AB_2737899	1
CD8a	BV510	RPA-T8	Biolegend	301048	RRID:AB_2561942	1
CD45RA	BV605	HI100	Biolegend	304133	RRID:AB_11126164	0.625
CD4	BV711	OKT4	Biolegend	317439	RRID:AB_11219404	1
CLA	FITC	HECA-452	Biolegend	321306	RRID:AB_492898	4
IL-13	PE	JES10-5A2	Biolegend	501903	RRID:AB_315198	0.83
IL-22	PE/Cy7	22URTI	Thermo Fisher Scientific	25-7229-42	RRID:AB_10853346	0.5
CD103	APC	Ber-ACT8	Biolegend	350216	RRID:AB_2563907	2.5
IFNg	AlexaFlour700	4S.B3	Biolegend	502519	RRID:AB_528920	5
Fixable LiveDead	NearIR	n/a	Thermo Fisher Scientific	65-0865-14	-	1:1000 dilution

Suppl. Table 1: Composition of (A) 20-color multiparameter spectral cytometry panel for immunophenotyping, and (B) antibody panel used for intracellular cytokine staining.

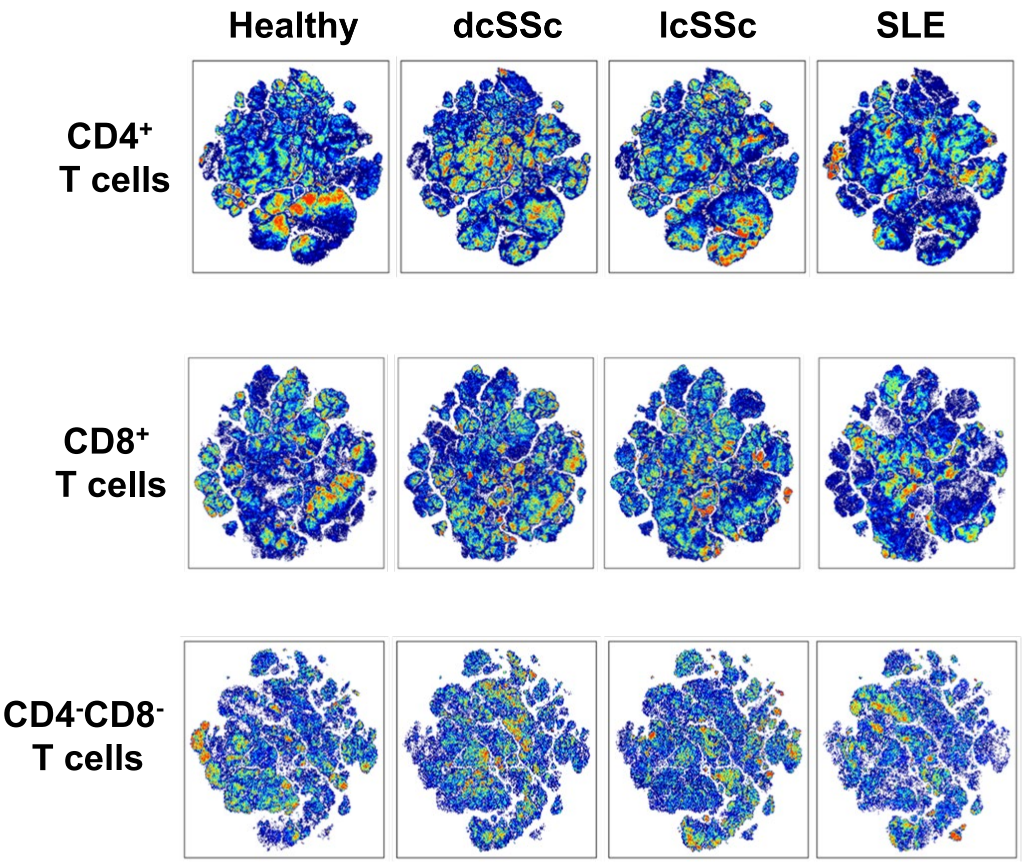

Suppl. Figure 1: Cell distribution comparison between healthy subjects, and dcSSc, lcSSc and SLE patients

Opt-SNE maps of CD4+, CD8+ and $CD4^{-}CD8^{-}$ T cells overlaid with cell densities for healthy controls, dcSSc, lcSSc and SLE patients. Each cohort was normalized to contain the same number of cells per cohort for each T cell lineage: $1x10^{6}$ cells for CD4+ and CD8+ T cells, and 2.5 x 10^{5} for $CD4^{-}CD8^{-}$ T cells. Data represents all samples concatenated.

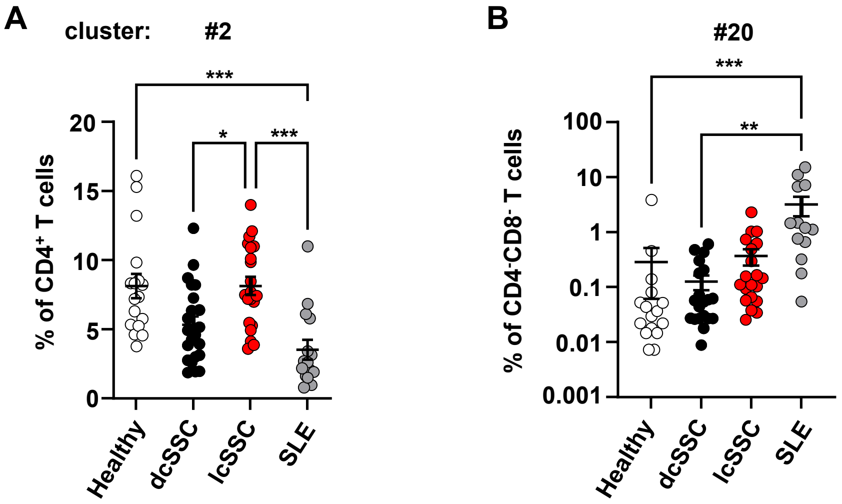

Suppl. Figure 2: Aberrant features of circulating Tfh and Bcl-6⁺ T cells in SSc and SLE

Frequencies of (A) Tfh and (B) Bcl-6+ clusters within CD4+ (cluster 2) and CD4-CD8- (cluster 20) parental populations of healthy controls (n=17), dcSSc (n=22), lcSSc (n=21), SLE (n=15) patients as shown in Fig. 1D, F. Bar graphs show percentages of gated cells of indicated clusters with mean and SEM, calculated as in Fig. 2. Each symbol represents a blood sample from an individual donor. Data were statistically analyzed with the Kruskall-Wallis test followed by Dunn's multiple comparisons test. *p<0.05, **p<0.01, ***p<0.001

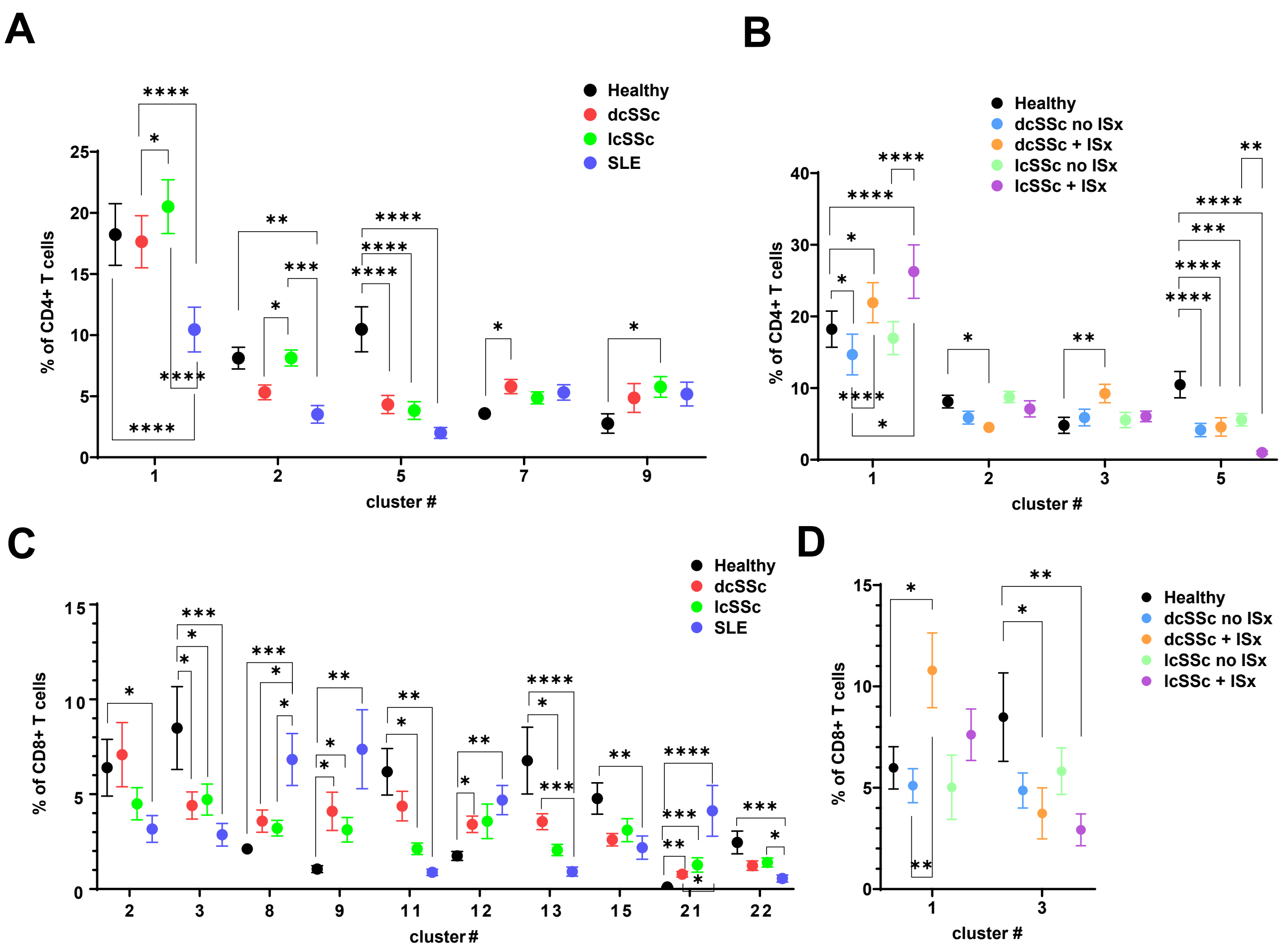

Suppl. Figure 3: Additional differences in frequencies of clusters not defined by transcription factor expression in peripheral blood

(A) Comparison of frequencies of selected clusters within the CD4+ T cell compartment identified in Fig. 1D and analyzed as in Fig. 2. Data is shown as the mean of healthy controls (n=17), dcSSc (n=22), lcSSc (n=21), and SLE (n=15) patients +/- SEM. (B) Impact of immunosuppressant treatment (ISx) on frequencies of selected clusters within CD4+ T cells. (C) Comparison of frequencies of selected clusters within the CD8+ T cell compartment identified in Fig. 1E and analyzed as in Fig. 2. (D) Impact of immunosuppressant treatment (ISx) on frequencies of selected clusters within CD8+ T cells. Data were statistically analyzed with two-way ANOVA test. *p<0.05, **p<0.01, ***p<0.001, ****p<0.0001

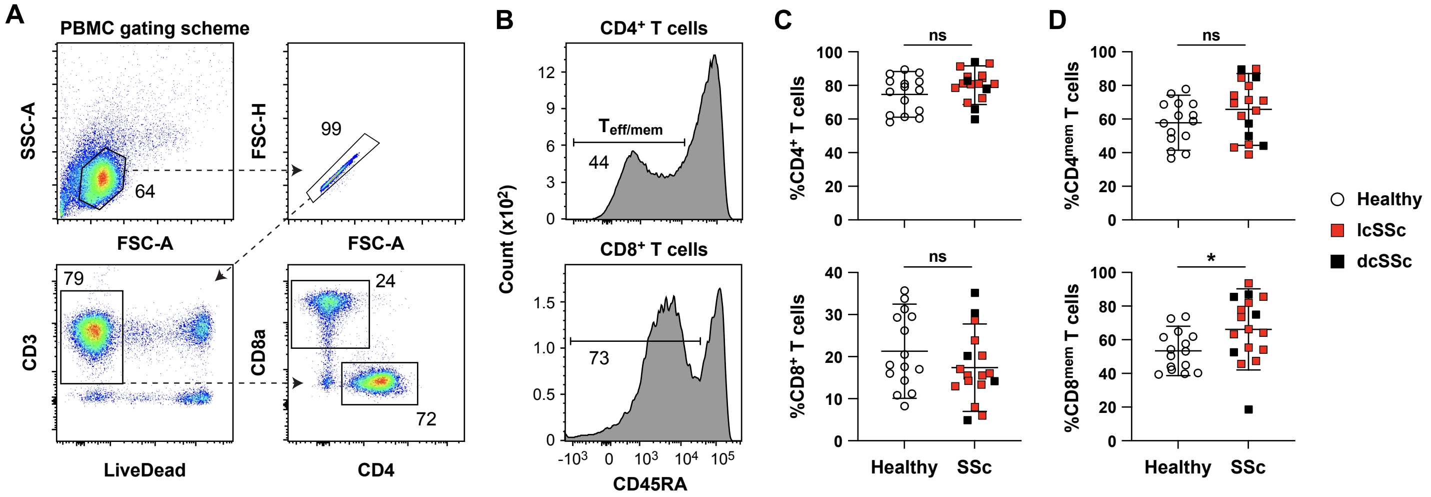

Suppl. Figure 4: **Gating strategy for peripheral blood T cells from SSc subjects.** (A) Flow cytometry gating for identification of CD3+CD4+ and CD3+CD8+ T cells from PBMCs, including singlet, viability, and lineage gating steps. (B) Representative CD45RA histograms for CD4+ and CD8+ T cells showing memory (CD45RAlo) versus naïve populations. (C-D) Frequencies of peripheral T cell subsets in healthy donors and SSc patients. (C) Proportion of CD4+ and CD8+ T cells among CD3+ T cells. (D) Percentage of CD45RAlo memory T cells among CD4+ (top) and CD8+ (bottom) subsets.

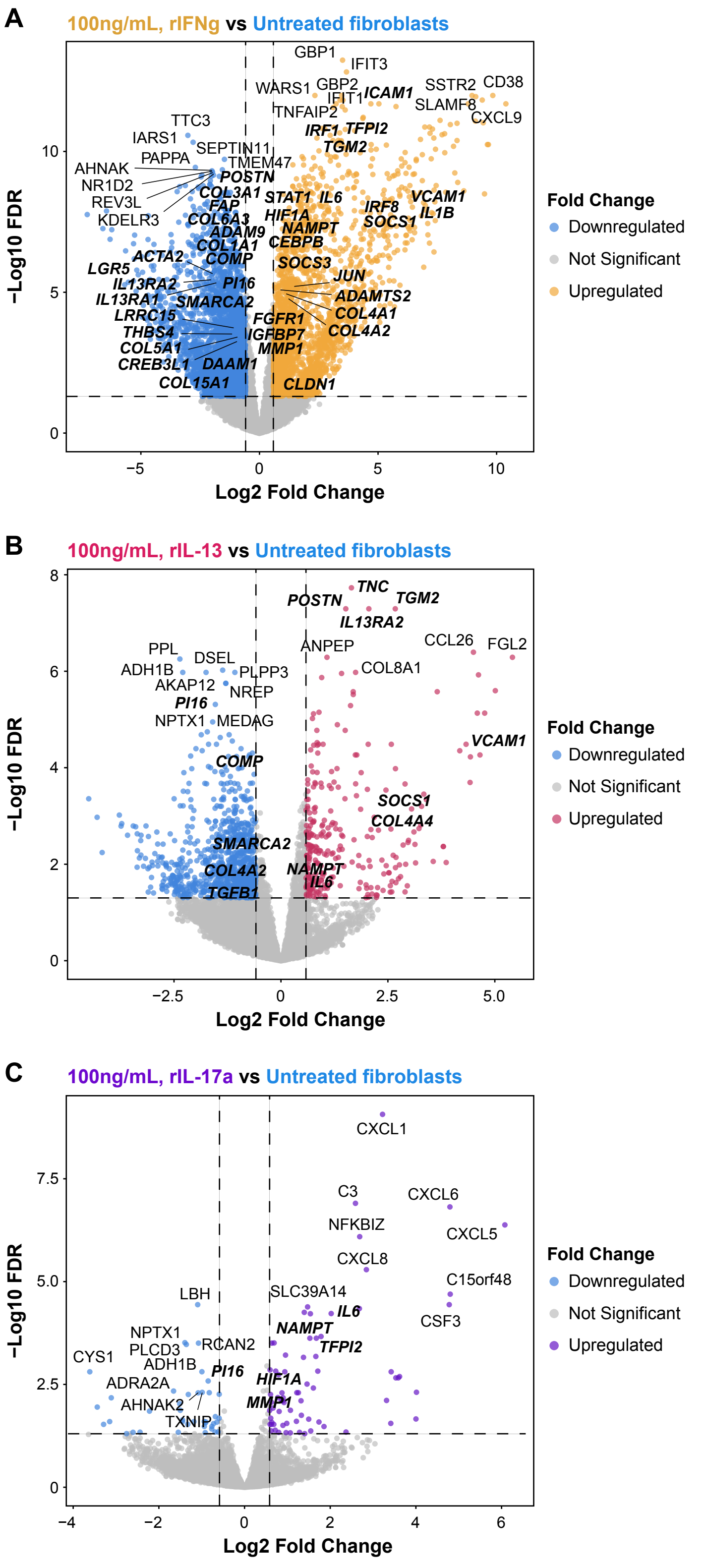

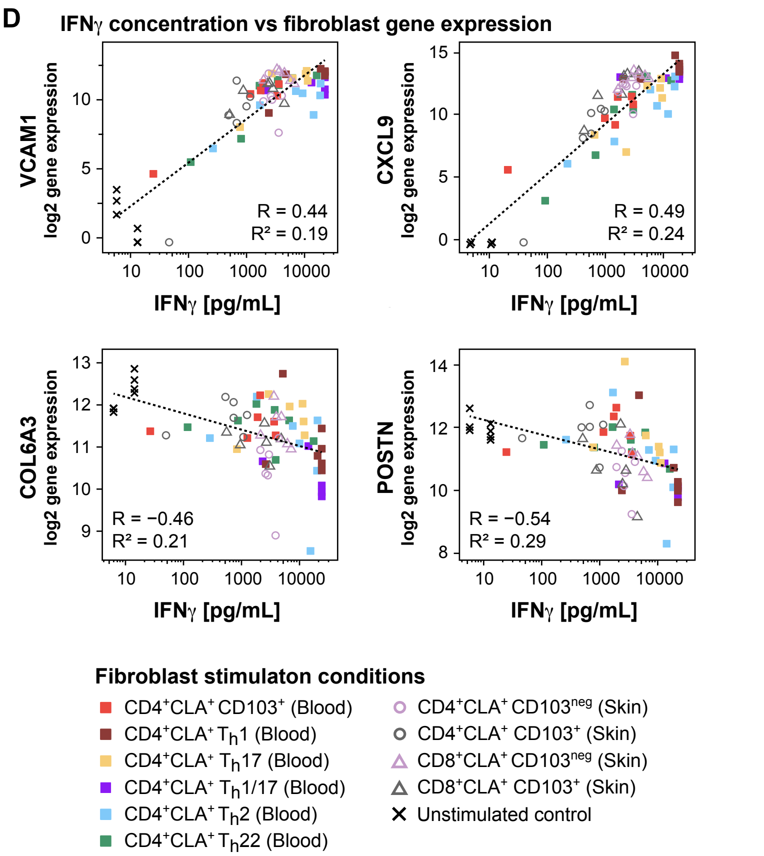

E **Multivariable model of IFNγ association with gene expression**

gene	*β (effect size)*	*p value*	*independent association*
CXCL9	3.33	2.86e-08	**strong positive association**
VCAM1	0.90	0.045	moderate positive association
THBS1	0.60	0.073	no independent association
COL1A1	0.13	0.698	no independent association
COL15A1	-0.41	0.217	no independent association
ACTA2	-0.53	0.074	no independent association
COL6A3	-0.72	0.014	moderate negative association
LGR5	-0.95	0.035	moderate negative association
POSTN	-1.25	0.001	**strong negative association**

Suppl. Figure 5: **Cytokine-specific gene regulation in dermal fibroblasts** (A-C) Volcano plots showing differentially expressed genes in fibroblasts treated for 24 hours with 100 ng/mL recombinant IFN-γ (A), IL-13 (B), or IL-17a (C), compared to untreated fibroblasts. The top 10 genes up- or down-regulated per condition ranked by -log10 FDR are shown. Bold italicized genes are differentially regulated and overlap with the heatmap in Figure 6D. Statistical significance: *p < 0.05, **p < 0.01, ns = not significant (unpaired t test or Mann-Whitney U test based on Shapiro-Wilk distribution testing). Error bars show mean ± SD. (D-E) Analysis of public data (ref 33).(D) Scatter plots showing the relationship between dermal fibroblast gene expression and IFN-γ concentration in human T cell supernatants (n=69). Each point represents an individual primary fibroblast culture stimulated with supernatant from *ex vivo* activated T cell populations. IFN-γ concentrations are displayed on a log10 scale. Dashed lines indicate linear regression fits. Pearson correlation coefficients R and R^2 values are shown. (E) Summary of multivariable linear modeling for cytokine associations with fibroblast gene expression. Each β value represents the change in gene expression per 10-fold increase in IFN-γ concentration after accounting for 12 other measured T cell cytokines. The p values indicate statistical significance of each association (two-sided, nominal). Independent association categories reflect direction and magnitude of β and statistical significance.
